# The gut microbiota metabolite capsiate promotes Gpx4 expression by activating *TRPV1* to inhibit intestinal ischemia reperfusion-induced ferroptosis

**DOI:** 10.1080/19490976.2021.1902719

**Published:** 2021-03-28

**Authors:** Fan Deng, Bing-Cheng Zhao, Xiao Yang, Ze-Bin Lin, Qi-Shun Sun, Yi-Fan Wang, Zheng-Zheng Yan, Wei-Feng Liu, Cai Li, Jing-Juan Hu, Ke-Xuan Liu

**Affiliations:** Department of Anesthesiology, Nanfang Hospital, Southern Medical University, Guangzhou, Guangdong, China

**Keywords:** Intestinal ischemia/reperfusion injury, metabolites, capsiate, ferroptosis, Gpx4, TRPV1

## Abstract

Ferroptosis, a new type of cell death has been found to aggravate intestinal ischemia/reperfusion (I/R) injury. However, little is known about the changes of gut microbiota and metabolites in intestinal I/R and the role of gut microbiota metabolites on ferroptosis-induced intestinal I/R injury. This study aimed to establish a mouse intestinal I/R model and ileum organoid hypoxia/reoxygenation (H/R) model to explore the changes of the gut microbiota and metabolites during intestinal I/R and protective ability of capsiate (CAT) against ferroptosis-dependent intestinal I/R injury. Intestinal I/R induced disturbance of gut microbiota and significant changes in metabolites. We found that CAT is a metabolite of the gut microbiota and that CAT levels in the preoperative stool of patients undergoing cardiopulmonary bypass were negatively correlated with intestinal I/R injury. Furthermore, CAT reduced ferroptosis-dependent intestinal I/R injury in vivo and in vitro. However, the protective effects of CAT against ferroptosis-dependent intestinal I/R injury were abolished by RSL3, an inhibitor of glutathione peroxidase 4 (Gpx4), which is a negative regulator of ferroptosis. We also found that the ability of CAT to promote Gpx4 expression and inhibit ferroptosis-dependent intestinal I/R injury was abrogated by JNJ-17203212, an antagonist of transient receptor potential cation channel subfamily V member 1 (TRPV1). This study suggests that the gut microbiota metabolite CAT enhances Gpx4 expression and inhibits ferroptosis by activating TRPV1 in intestinal I/R injury, providing a potential avenue for the management of intestinal I/R injury.

## Introduction

The intestine plays an important role in maintaining physiological homeostasis and activities, such as nutrient absorption, toxin excretion, maintenance of immune homeostasis, and hormone release.^1^ However, several fatal diseases, such as traumatic shock, severe infection, and severe burns, can cause intestinal ischemia/reperfusion (I/R) injury.^2^ Compared with the local injury that occurs during intestinal ischemia, the intestinal flora displacement and massive release of endotoxins and inflammatory factors that occur during blood reperfusion often leading to intestinal and intestinal distal organ (lung, liver, kidney, heart, brain, etc.) failure and even death.^3,4^ Therefore, studying the mechanism and potential treatment strategies for intestinal I/R injury; and looking for biomarkers that can effectively predict the risk of intestinal I/R injury in perioperative patients for early prevention are very valuable to reduce the high prevalence and mortality of intestinal I/R injury.

The differences in gut microbiota and its metabolites have recently been shown to play an important role in disease prediction, diagnosis, and treatment.^5^ The changes in gut microbiota induced by intestinal I/R have been reported many years ago,^6,7^ but the application of new sequencing technologies and the role of specific bacterial metabolites in intestinal I/R injury have not yet been elucidated. In this study, 16S rRNA gene sequencing and metabolomics were performed to explore the changes in the gut microbiota and metabolites in intestinal I/R injury. Then we found that capsiate (CAT) is a metabolite of the gut microbiota and was drastically reduced by intestinal I/R by 2–3 times. CAT is an oily colorless substance derived from the Solanaceae plant sweet pepper. CAT has been found to promote energy consumption and metabolism,^8^ inhibit fat accumulation,^9^ antioxidative,^8^ anti-inflammatory,^10^ and antitumor properties.^11^ *TRPV1* has been proven to be the most important receptor for CAT to perform multiple biological functions.^12,13^ However, which role CAT plays in intestinal I/R injury has not yet been determined.

Ferroptosis is a type of cell death that is dependent on ferrous ions (Fe^2+^) and is caused by the accumulation of lipid peroxides and reactive oxygen species.^14,15^ Intracellular redox homeostasis is disrupted in the I/R process. Furthermore, ferroptosis has been observed in I/R injury and has been found to aggravate I/R injury.^16^ Glutathione peroxidase 4 (Gpx4) is important for maintaining lipid redox homeostasis and is a negative regulator of ferroptosis.^17^ As mentioned, CAT exhibits resistance to lipid peroxides^18^ and oxidative stress,^8^ which are the two key factors of ferroptosis. However, whether CAT can reduce ferroptosis in intestinal I/R injury is currently unclear.

Therefore, this study was performed to explore the changes in the gut microbiota and metabolites in intestinal I/R injury, and determine the role and mechanism by which CAT protects against ferroptosis-related intestinal I/R injury. Furthermore, we aimed to explore the possibility of gut microbiota metabolite-CAT reducing intestinal I/R injury and to provide a theoretical basis for CAT treatment of intestinal I/R injury.

## Results

### Ferroptosis is present in and enhances intestinal I/R injury

**Figure 1.**
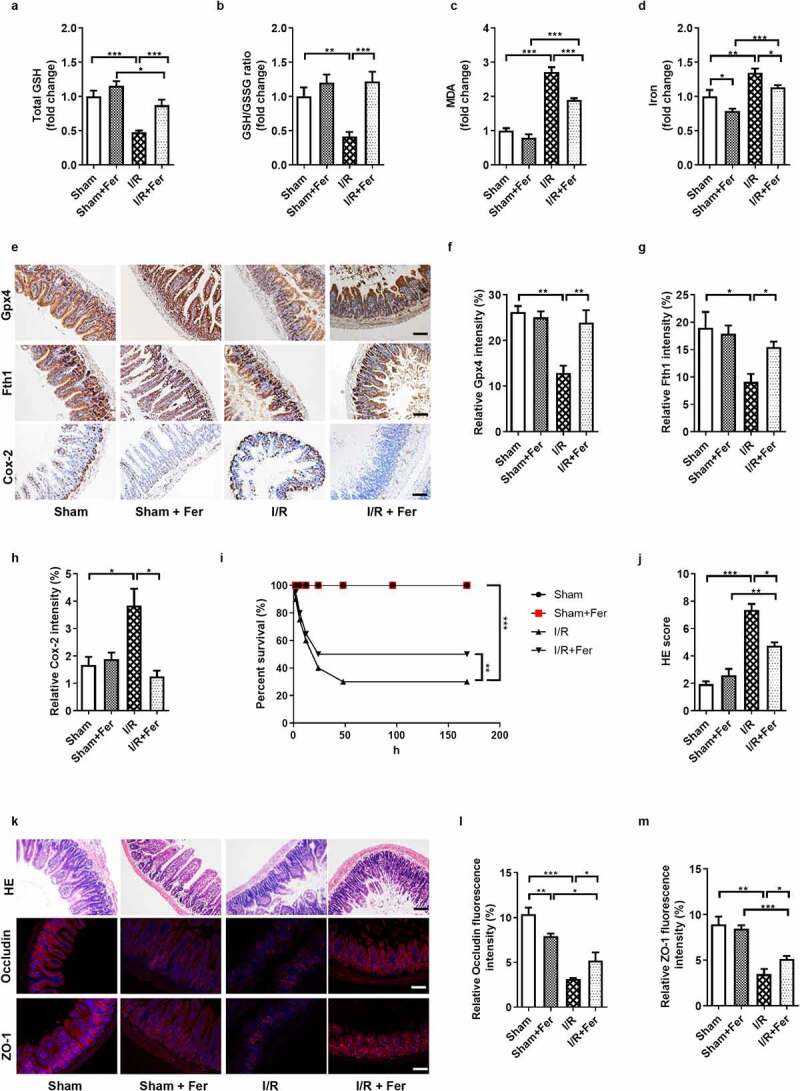
**Ferroptosis is present in and enhances intestinal ischemia/reperfusion (I/R) injury in mice**. (a-b) The total glutathione (GSH) and GSH/GSSG levels in the intestinal tissue. (c-d) Malondialdehyde (MDA) and Fe^2+^ levels in the intestinal tissue. (e) The protein levels of glutathione peroxidase 4 (Gpx4), ferritin heavy chain 1 (Fth1) and cyclooxygenase 2 (Cox-2), scale bar is 100 μm. (f-h) Relative quantitative statistics of Gpx4, Fth1 and Cox-2 protein expression. (i) 7-day survival rate of mice after intestinal I/R, n = 20. (j) Chiu’s pathology score. (k) Hematoxylin-eosin (HE) staining of small intestine tissue and the relative protein levels of the intestinal barrier tight junction Occludin and zona occludens (ZO)-1, scale bar is 100 μm. (l-m) Relative fluorescence quantitative statistics of Occludin and ZO-1 protein expression. The results are expressed as the mean ± SEM. n = 8. * *p* < .05, ** *p* < .01, *** *p* < .001 by one-way ANOVA (Tukey’s test)

Perform a midline laparotomy on the anesthetized mice to identify the superior mesenteric artery of the mice, block it with microvascular clamps for 1 h, and then perfusion for 2 h. Then ferroptosis inhibitor ferrostatin-1 (Fer) were used to explore the role of ferroptosis played in intestinal I/R injury. The total GSH (Figure 1a) and total GSH/GSSG ratio (Figure 1b) were lower, while MDA (Figure 1c) and Fe^2+^ (Figure 1d) levels were higher in the I/R group than in the sham group, and there was no significant difference in total GSH, total GSH/GSSG ratio, MDA levels between Sham group and Sham + Fer group. Meanwhile, the mRNA and protein levels of ferroptosis-negative regulators Gpx4 and Fth1 were downregulated, while the mRNA and protein levels of the ferroptosis marker Cox-2 were upregulated by intestinal I/R (Figure 1(e-h)), Fig. S1C-E). These data indicate that ferroptosis is present in intestinal I/R injury. Induced intestinal I/R decreased the 7-day survival rate of mice (Figure 1i) and increased small intestinal mucosal damage (Figure 1(j-k)). Furthermore, intestinal I/R disrupted the intestinal barrier homeostasis characterized by reduced relative mRNA and protein levels of tight junction Occludin and ZO-1 (Figure 1(k-m), Fig. S1F-G). All these data changes were mitigated by the ferroptosis inhibitor Fer under I/R condition.

### Ferroptosis is present in and enhances intestinal organoid H/R injury in vitro

**Figure 2.**
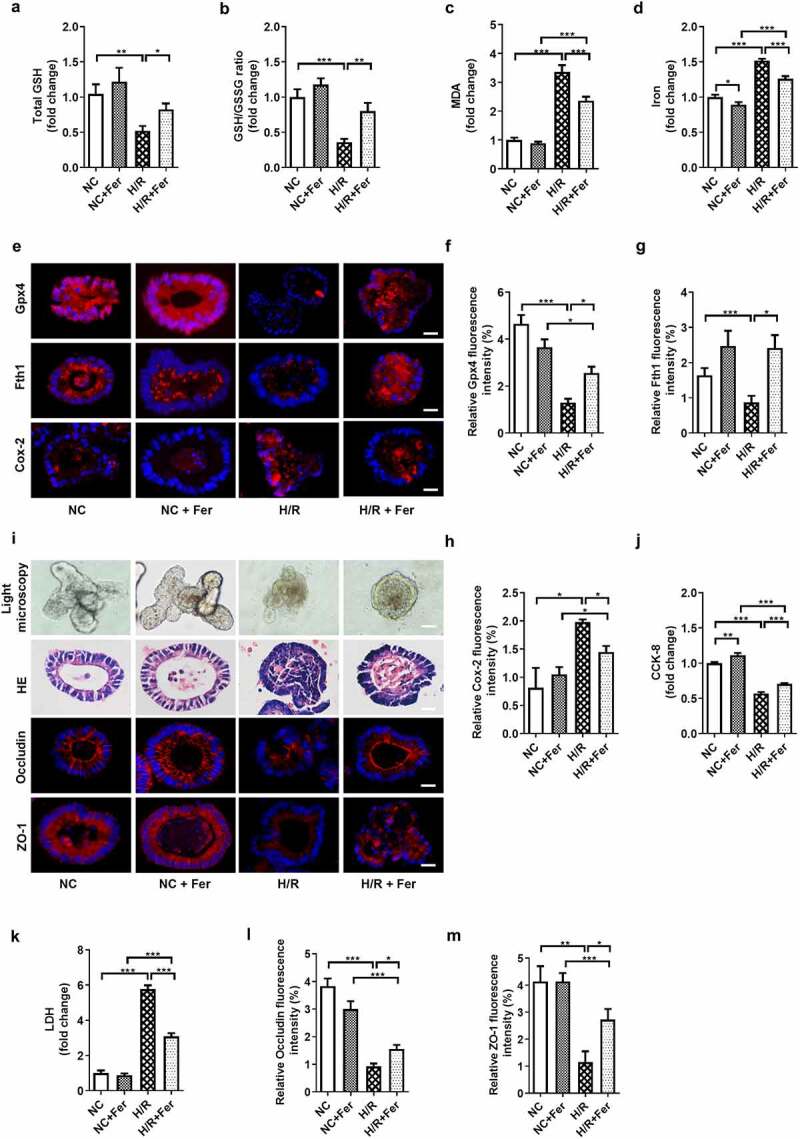
**Ferroptosis is present in and enhances intestinal organoid H/R injury**. (a-b) Total GSH and the ratio of total GSH/GSSG in the organoids. (c-d) MDA and Fe^2+^ levels in the organoids. (e) The protein levels of Gpx4, Fth1 and Cox-2 in the organoids, scale bar is 20 μm. (f-h) Relative fluorescence quantitative statistics of Gpx4, Fth1 and Cox-2 protein expression. (i) Ileum organoid morphology observed by light microscopy and HE staining and the expression of Occludin and ZO-1 in the organoids observed by immunofluorescence, scale bar is 20 μm. (j) Organoid viability measured by CCK-8. (k) Levels of lactate dehydrogenase (LDH) released into the medium. (l-m) Relative fluorescence quantitative statistics of Occludin and ZO-1 protein expression in the organoids. The results are expressed as the mean ± SEM. n = 6. * *p* < .05, ** *p* < .01, *** *p* < .001 by one-way ANOVA (Tukey’s test)

Establish a mouse small intestinal organoids H/R model to observe ferroptosis of organoids, and then Fer were used to explore the role of ferroptosis played in organoids H/R injury. Total GSH (Figure 2a) and the total GSH/GSSG ratio (Figure 2b) were lower, while MDA (Figure 2c) and Fe^2+^ (Figure 2d) levels were higher in the H/R group than in the NC group, and there was no significant difference in total GSH, total GSH/GSSG ratio, MDA and Fe^2+^ levels between NC group and NC + Fer group. Meanwhile, the mRNA and protein levels of Gpx4 and Fth1 were downregulated, while the mRNA and protein levels of Cox-2 were upregulated by organoid H/R (Figure 2(e-h), Fig. S1H-J). In addition, small intestinal organoid H/R destroyed the normal morphological structure of organoids, as observed under the light microscope (Figure 2i), leading to pathological damage of organoids, as determined by HE staining (Figure 2i), and accompanied by reduced organoid activity (Figure 2j) and increased levels of LDH released into the medium (Figure 2k). Furthermore, organoid H/R disrupted intestinal barrier homeostasis as characterized by reduced relative mRNA and protein levels of tight junction Occludin and ZO-1 (Figure 2(l-m), Fig. S1K-L). These data changes were mitigated by Fer under H/R condition.

### Intestinal I/R-induced changes in the gut microbiota and its metabolites

**Figure 3.**
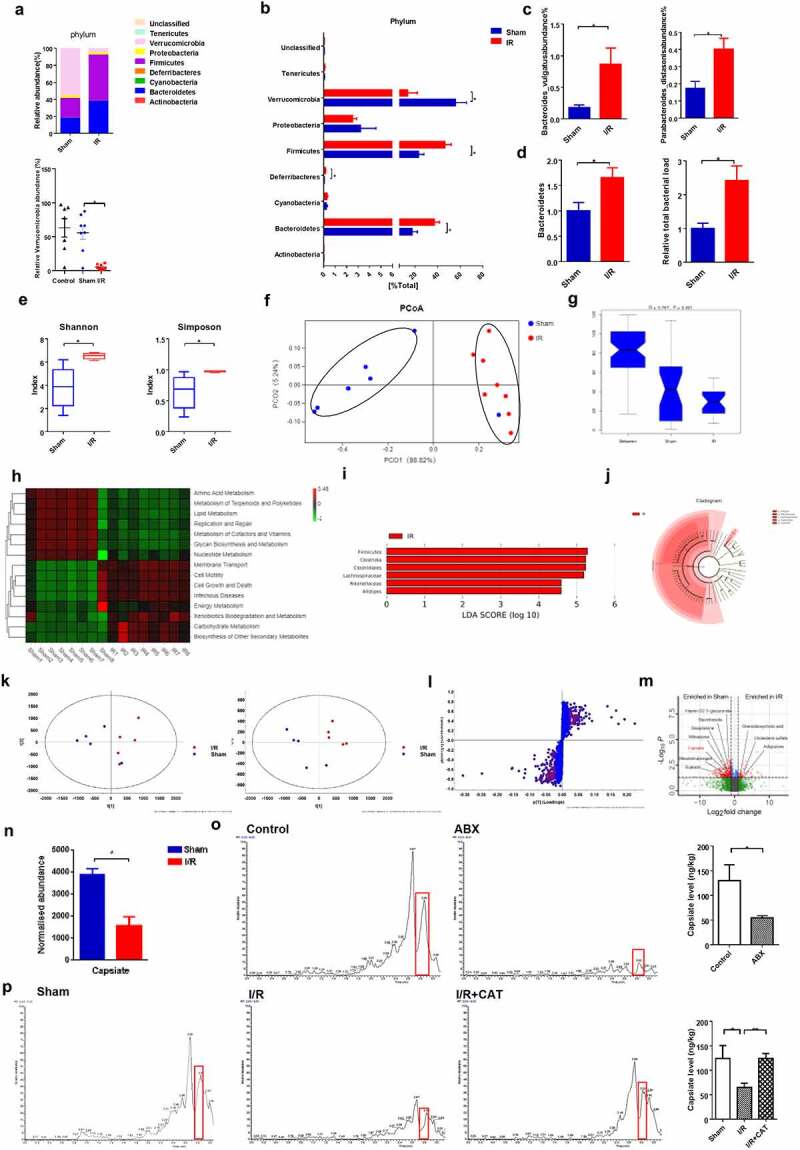
**Intestinal I/R-induced changes in the gut microbiota and its metabolites**. (a-b) Relative bacterial abundance at the phyla level in the cecum of mice. (c) Relative abundance of *Bacteroides vulgatus* and *Parabacteroides distasonis* at the species level in the cecum. (d) Relative amounts of Bacteroidetes and total bacterial load in the cecum by qPCR. (e) Alpha diversity indices (Shannon and Simpson). (f) PCoA based on the unweighted UniFrac analysis of operational taxonomic units (OTUs). (g) Anosim analysis on unweighted UniFrac distances in the fecal microbiota between the Sham and I/R mice. (h) Heatmap of the expression values of signaling pathways in each sample based on Tax4Fun analysis. The expression values of 16 samples are presented as the normalized z-score using the enrichment scores of signaling pathways. (i-j) Histogram of the LDA score showing the enriched bacteria and taxa in the gut microbiome of the I/R mice represented in the cladogram. (k-l) Scatter plots of PCA and OPLS-DA and S‐Plot analysis of metabolomics of cecal content. (m) Volcano plot showing the differentially accumulated [log_2_ (fold change) on X-axis] and significantly changed [-log_10_ (p) on Y-axis] metabolites in the Sham and I/R group. (n) CAT levels in cecum (nontargeted), (o) CAT levels after ABX treatment in cecum (P) CAT levels in cecum (targeted) from sham, I/R and I/R + CAT mice. The results are expressed as the mean ± SEM. n = 6–8. * *p* < .05, ** *p* < .01, *** *p* < .001 by one-way ANOVA (Tukey’s test)

First, PCR and 16S rRNA gene sequencing were performed to explore the changes in the gut microbiota in intestinal I/R injury. 16S rRNA sequencing showed a significant increase in the relative abundance of *Firmicutes* and *Bacteroidetes* as well as a significant decrease in the relative abundance of *Verrucomicrobia* in the I/R group compared with the sham group, at the phylum level. Compared with the untreated control group, the operation of sham group had no significant effect on the relative abundance of *Verrucomicrobia* (Figure 3(a-b)). In addition, the relative abundance of *Bacteroides vulgatus* and *Parabacteroides distasonis* at the species level were increased in the I/R group compared with the sham group (Figure 3c). Meanwhile, PCR results also showed that the relative abundance of *Bacteroidetes* and the relative total bacterial load in the cecum content significantly increased in the I/R group compared with the sham group (Figure 3d). Furthermore, the I/R group showed a significantly higher bacterial diversity (Shannon, *p* = .004; Simpson, *p* = .006; Wilcoxon test) than the sham group (Figure 3e). PCA and analysis of similarity (Anosim) on unweighted UniFrac distances were performed and indicated that the gut microbiota of the sham and I/R groups had completely separate clusters (figure 3(f-g)). In particular, Tax4Fun prediction analysis showed that the genomic abundance of some pathways, such as those for the biosynthesis of secondary metabolites and glycan biosynthesis and metabolism were significantly impaired in the I/R group compared to the sham group (Figure 3h). Linear discriminant effect size (LEfSe) analysis and cladograms were used to analyze the fecal bacterial signature profiles and predominant bacterial biomarker (Figure 3(i-j)). Taken together, these data clearly indicated that the intestinal microbiota have different diversity in the Sham and I/R mice.

Next, the microflora untargeted metabolomics was performed to further explore the function of intestinal microflora metabolites in intestinal I/R injury. Differential metabolites were identified as the variables with *p* < .05 and variable importance in projection (VIP) >1 (Table S1). PCA and OPLS-DA showed that the metabolite profiles were different between the two groups (Figure 3k). The metabolites capsiate measured by mass spectrometer is identified in public databases HMDB and Mass Bank based on m/z value and retention time (RT), its molecular formula is C18H26O4 and adducts are M + H, M+ Na. S‐Plot analysis and volcano plot showed that the differential metabolites represented by CAT in the Sham and I/R group (Figure 3(l-m)), as well as the CAT levels in cecum was statistically decreased in the I/R group compared with the sham group (nontargeted) (Figure 3n) Taking into account factors such as the significantly changing multiple levels of metabolites, the correlation between metabolites and postoperative intestinal I/R injury in patients and whether their effects and functions have the potential to reduce intestinal I/R damage, CAT then entered our field of vision. Targeted metabolomic analysis was used to further verify that the gut microbiota contributes to CAT levels. The results of targeted metabolism show that there was no CAT content in the diet of mice (Fig. S2A-B). Then the mice were treated with antibiotics (ABX) (vancomycin, 100 mg/kg; neomycin sulfate 200 mg/kg; metronidazole 200 mg/kg; and ampicillin 200 mg/kg) intragastrically once each day for 1 week to deplete the gut microbiota,^19,20^ which resulted in a significant decrease in relative total bacterial load in the feces and in the CAT levels in the cecum relative to those in the control mice, as determined by LC-MS/MS (Figure 3o and Fig. S2C). In addition, the CAT level in the I/R group was significantly reduced compared with that in the sham group, but I/R + CAT treatment significantly reversed this trend (Figure 3p). Our data clearly demonstrated that the gut microbiota can generate CAT. Correlation analysis results show that there is no obvious correlation between the relative abundance of *Verrucomicrobia* and the level of capsiate in cecum (Fig. S2D). Therefore, the changes of CAT levels in the cecum may not be related to the relative abundance of *Verrucomicrobia*.

#### CAT inhibits I/R-induced ferroptosis

**Figure 4.**
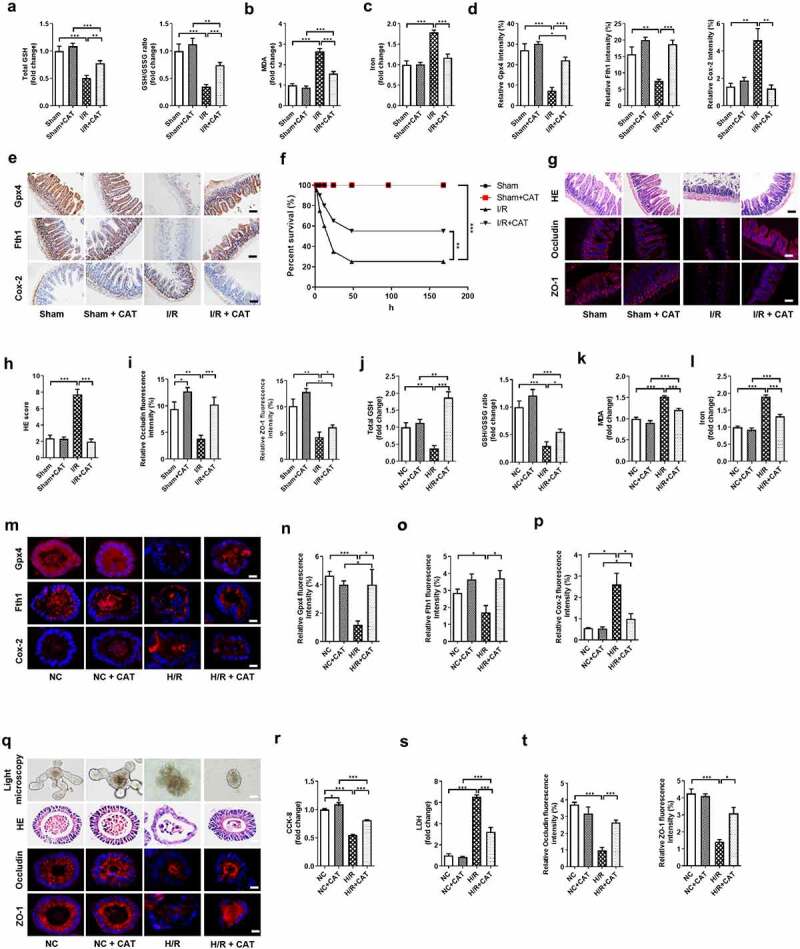
**CAT inhibits intestinal I/R-induced ferroptosis**. (a) The total GSH and GSH/GSSG levels in the intestinal tissue. (b-c) MDA and Fe^2+^ levels in the intestinal tissue. (d) Relative quantitative statistics of Gpx4, Fth1 and Cox-2 protein expression. (e) The protein levels of glutathione Gpx4, Fth1 and Cox-2, scale bar is 100 μm. (f) 7-day survival rate of mice after intestinal I/R, n = 20. (g) HE staining of small intestine tissue and the relative protein levels of Occludin and ZO-1, scale bar is 100 μm. (h) Chiu’s pathology score. (i) Relative fluorescence quantitative statistics of Occludin and ZO-1 protein expression. (j) Total GSH and the ratio of total GSH/GSSG in the organoids. (k-l) MDA and Fe^2+^ levels in the organoids. (m) The protein levels of Gpx4, Fth1 and Cox-2 in the organoids, scale bar is 20 μm. (n-p) Relative fluorescence quantitative statistics of Gpx4, Fth1 and Cox-2 protein expression. (q) Ileum organoid morphology observed by light microscopy and HE staining and the expression of Occludin and ZO-1 in the organoids observed by immunofluorescence, scale bar is 20 μm. (r) Organoid viability measured by CCK-8. (s) LDH released into the medium. (t) Relative fluorescence quantitative statistics of Occludin and ZO-1 protein expression in the organoids. The results are expressed as the mean ± SEM. n = 8 *in vivo*; n = 6 *in vitro*. * *p* < .05, ** *p* < .01, *** *p* < .001 by one-way ANOVA (Tukey’s test)

Next, we observed the protective effect of CAT treatment on intestinal I/R injury in mice and organoids. Total GSH and the total GSH/GSSG ratio were higher (Figure 4a), while MDA (Figure 4b) and Fe^2+^ (Figure 4c) levels were lower in the I/R + CAT group than in the I/R group, and there was no significant difference in these indicators between Sham group and Sham + CAT group. Meanwhile, the mRNA and protein levels of Gpx4 and Fth1 were upregulated, while the mRNA and protein levels of Cox-2 was downregulated by CAT under I/R condition, but not in Sham condition (Figure 4(d-e), Fig. S2G-I). Compared to the I/R group, the survival rate of mice significantly increased, while epithelial damage, as detected by HE staining, was reduced in the I/R + CAT group (figure 4(f-h)). Furthermore, the relative mRNA and protein levels of tight junction Occludin and ZO-1 in the I/R + CAT group were higher than those in the I/R group (Figure 4(g, i); Fig. S2J-K).

Consistent with the in vivo results, total GSH and total GSH/GSSG ratio were higher (Figure 4j), while MDA (Figure 4k) and Fe^2+^ (Figure 4l) levels were lower in the H/R + CAT group than those in the H/R group, and there was no significant difference in these indicators between NC group and NC + CAT group. Moreover, the mRNA and protein levels of Gpx4 and Fth1 were upregulated, while the mRNA and protein levels of Cox-2 were downregulated by CAT under H/R condition, but not in NC condition (Figure 4(m-p), Fig. S2L-N). CAT treatment mitigated organoid morphological and pathological injury (Figure 4q), promoted organoid vitality (Figure 4r), and reduced the levels of LDH (Figure 4s) induced by H/R. CAT treatment also increased the relative mRNA and protein levels of tight junction Occludin and ZO-1 under H/R condition (Figure 4q, t; Fig. S2O-P).

### CAT inhibits ferroptosis-dependent intestinal I/R injury by promoting Gpx4 expression

**Figure 5.**
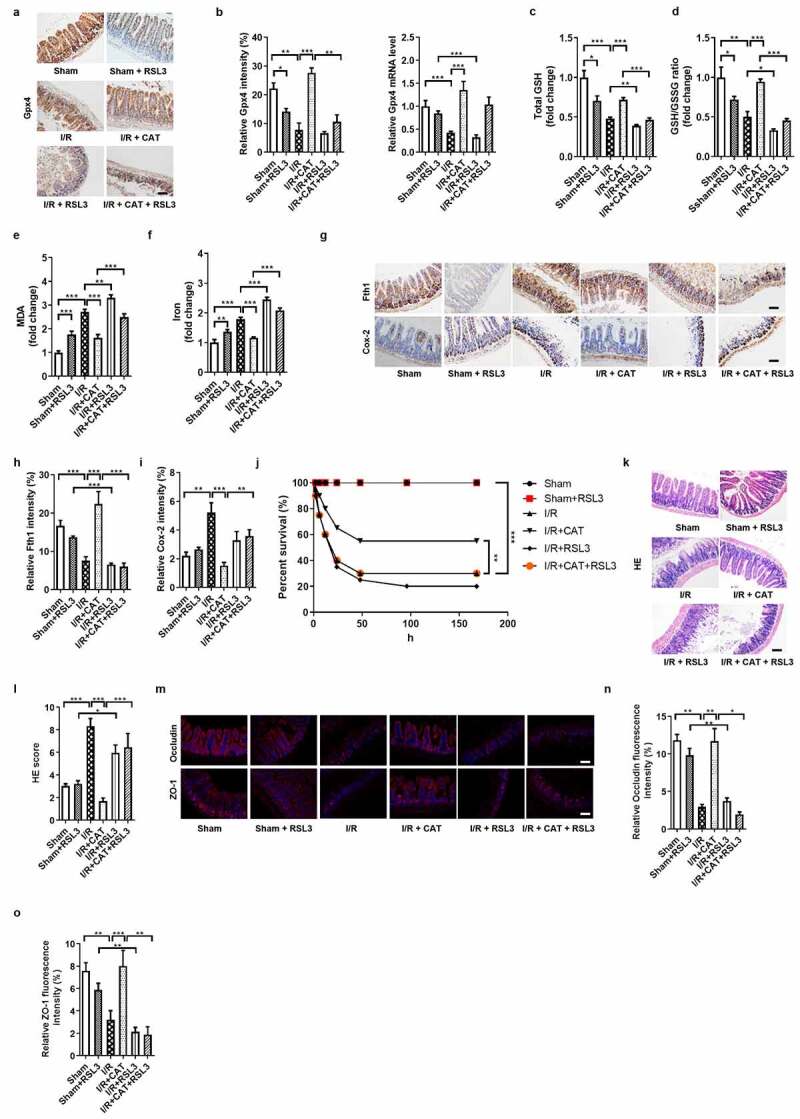
**CAT inhibits ferroptosis-dependent intestinal I/R injury by promoting Gpx4 expression**. (a) The protein levels of glutathione Gpx4, scale bar is 100 μm. (b) Relative quantitative statistics of Gpx4 protein expression and relative Gpx4 mRNA level in the intestinal tissue. (c-d) The total glutathione (GSH) and GSH/GSSG levels in the intestinal tissue. (e-f) Malondialdehyde (MDA) and Fe^2+^ levels in the intestinal tissue. (g) The protein levels of Fth1 and Cox-2, scale bar is 100 μm. (h-i) Relative quantitative statistics of Fth1 and Cox-2 protein expression. (j) 7-day survival rate of mice after intestinal I/R, n = 20. (k-l) HE staining of small intestine tissue and Chiu’s pathology score. scale bar is 100 μm. (m) The relative protein levels of the intestinal barrier tight junction Occludin and ZO-1, scale bar is 100 μm. (n-o) Relative fluorescence quantitative statistics of Occludin and ZO-1 protein expression. The results are expressed as the mean ± SEM. n = 8. * *p* < .05, ** *p* < .01, *** *p* < .001 by one-way ANOVA (Tukey’s test)

We next confirmed the protectivity of CAT against intestinal I/R injury and the underlying mechanism using RSL3, a Gpx4 inhibitor.^21–23^ The protein levels of Gpx4 were upregulated by CAT, but were downregulated by RSL3 (Figure 5(a-b)). Compared with I/R + CAT group, total GSH and total GSH/GSSG ratio were decreased (Figure 5(c-d)), while MDA (Figure 5e) and Fe^2+^ (figure 5f) levels were increased by CAT + RSL3 treatment under I/R condition. Meanwhile, the mRNA and protein levels of Fth1 were downregulated, while the mRNA and protein levels of Cox-2 were upregulated by CAT + RSL3 during intestinal I/R (Figure 5(g-i), Fig. S3C-D). Compared to mice in the I/R + CAT group, mice in the CAT + RSL3 treatment group had significantly increased intestinal tissue pathological damage scores for HE staining and had reduced survival rates (Figure 5(j-l)). CAT + RSL3 also decreased the relative mRNA and protein levels of tight junction Occludin and ZO-1 (Figure 5(m-o), Fig. S3E-F).

### CAT inhibits ferroptosis-dependent intestinal organoid H/R injury by promoting Gpx4 expression in vitro

**Figure 6.**
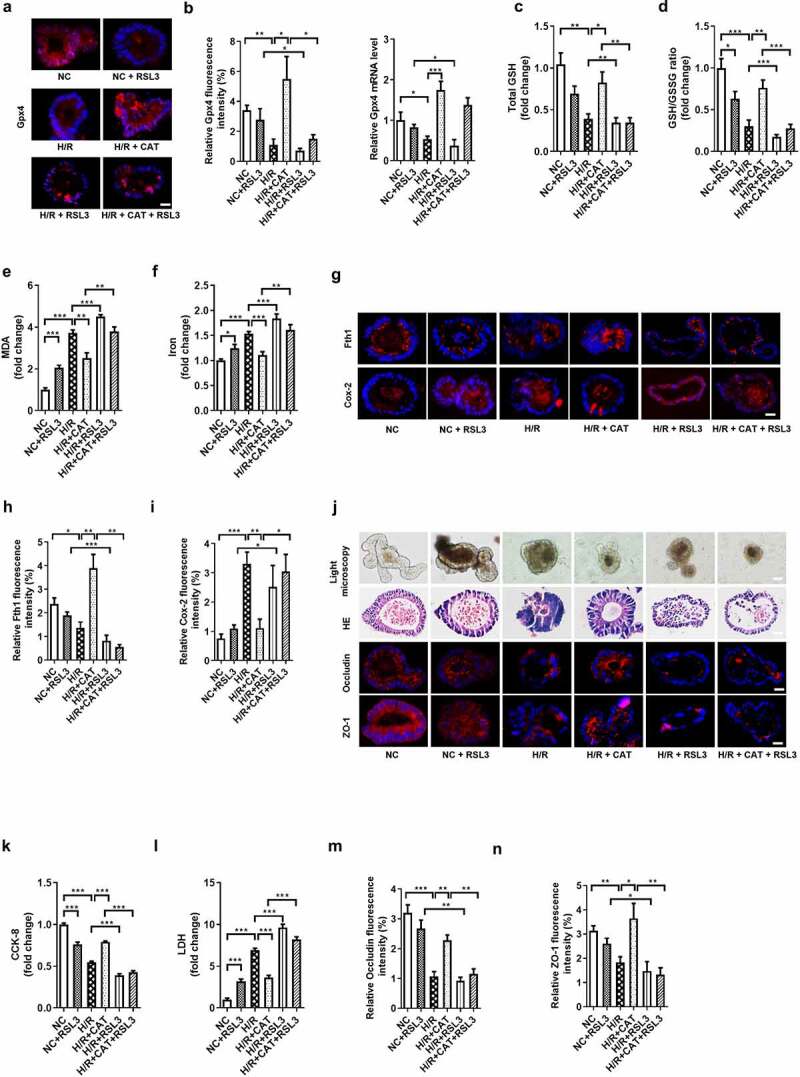
**CAT inhibits ferroptosis-dependent intestinal organoid H/R injury by promoting Gpx4 expression *in vitro***. (a)The protein levels of Gpx4 in the organoids, scale bar is 20 μm. (b) Relative fluorescence quantitative statistics of Gpx4 protein expression and relative Gpx4 mRNA level in the organoids. (c-d) Total GSH and the ratio of total GSH/GSSG in organoids. (e-f) MDA and Fe^2+^ levels in the organoids. (g) The protein levels of Fth1 and Cox-2, scale bar is 20 μm. (h-i) Relative fluorescence quantitative statistics of Fth1 and Cox-2 protein expression. (j) Ileum organoid morphology observed by light microscopy and HE staining and the expression of Occludin and ZO-1 in the organoids observed by immunofluorescence, scale bar is 20 μm. (k) Organoid viability measured by CCK-8. (l) Levels of LDH released into the medium. (m-n) Relative fluorescence quantitative statistics of Occludin and ZO-1 protein expression. The results are expressed as the mean ± SEM. n = 6. * *p* < .05, ** *p* < .01, *** *p* < .001 by one-way ANOVA (Tukey’s test)

We next confirmed the protectivity of CAT against organoids H/R injury and the underlying mechanism using RSL3, a Gpx4 inhibitor. The protein levels of Gpx4 were upregulated by CAT, but were downregulated by RSL3 during H/R (Figure 6(a-b)). Total GSH and total GSH/GSSG ratio were higher (Figure 6(c-d)), while the MDA and Fe^2+^ levels were lower in the H/R + CAT group than those in the H/R + CAT + RSL3 group (Figure 6(e-f)). Meanwhile, CAT treatment increased the mRNA and protein levels of Fth1 and decreased the mRNA and protein levels of Cox-2, which abolished by RSL3 during H/R (Figure 6(g-i), Fig. S3G-H). Small intestinal organoid H/R-induced in the H/R + CAT group was milder than in the H/R + CAT + RSL3 group, as indicated by light microscopy and HE staining (Figure 6j). Compared with the H/R + CAT group, organoid vitality decreased while LDH released in the medium increased in the H/R + CAT + RSL3 group (Figure 6(k-l)). Furthermore, CAT treatment increased the relative mRNA and protein levels of tight junction Occludin and ZO-1, which were downregulated by RSL3 during H/R (Figure 6(j, m-n); Fig. S3I-J).

### CAT enhances Gpx4 expression and inhibits ferroptosis by activating TRPV1 in intestinal I/R injury

**Figure 7.**
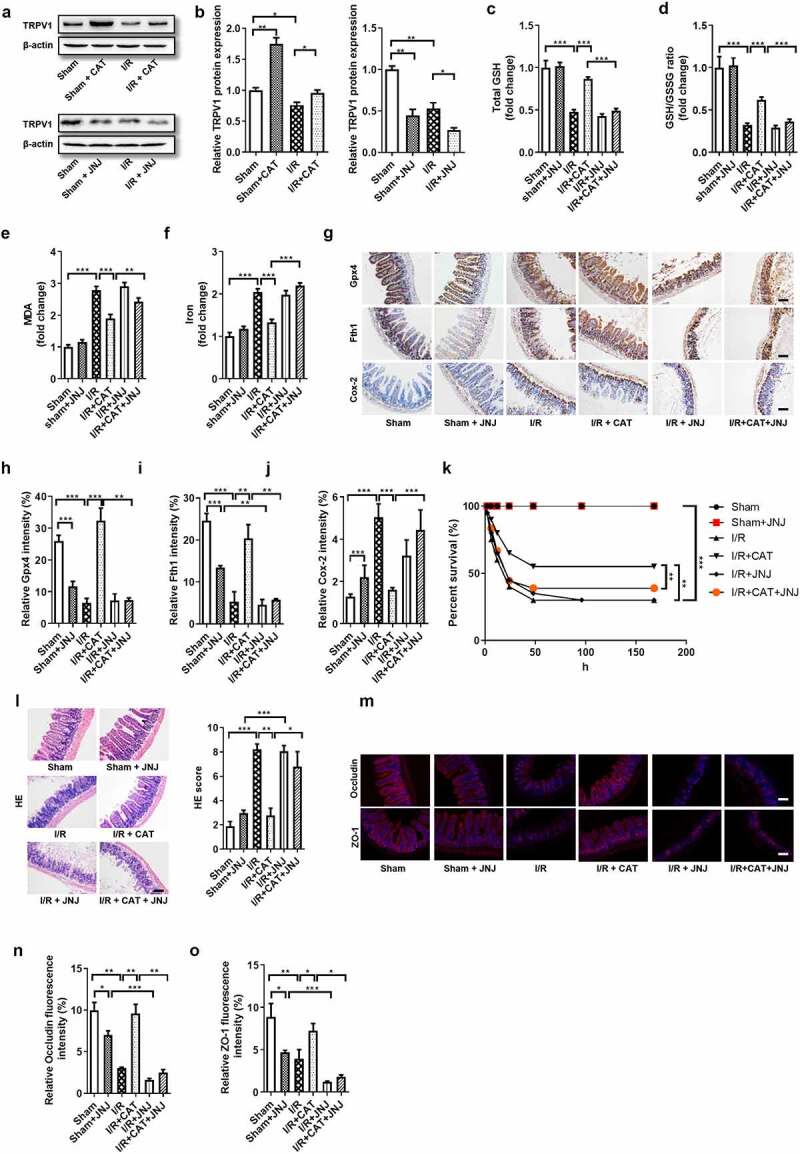
**CAT enhances Gpx4 expression and inhibits ferroptosis by activating TRPV1 in intestinal I/R injury**. (a-b) TRPV1 protein level in the ileum. (c-d) The total GSH and GSH/GSSG levels in the intestinal tissue. (e-f) MDA and Fe^2+^ levels in the intestinal tissue. (g) The protein levels of Gpx4, Fth1 and Cox-2, scale bar is 100 μm. (h-j) Relative quantitative statistics of Gpx4, Fth1 and Cox-2 protein expression (k) 7-day survival rate of mice after intestinal I/R, n = 20. (l) HE staining of small intestine tissue and Chiu’s pathology score, scale bar is 100 μm. (m-o) The protein levels of the Occludin and ZO-1 and relative fluorescence quantitative statistics of Occludin and ZO-1 protein expression., scale bar is 100 μm. The results are expressed as the mean ± SEM. n = 8. * *p* < .05, ** *p* < .01, *** *p* < .001 by one-way ANOVA (Tukey’s test)

CAT performs multiple functions depending on its activation of TRPV1.^12^ However, whether CAT promotes the expression of GPX4 and inhibits ferroptosis through *TRPV1* is still unclear. Therefore, the *TRPV1* antagonist JNJ-17203212 (JNJ) was used to further explore the role of the *TRPV1* in the protective ability of CAT against intestinal I/R-induced ferroptosis and injury. The mRNA and protein levels of *TRPV1* were upregulated by CAT, but were downregulated by JNJ (Figure 7(a-b), Fig. S4C). Consistent with the previous results, CAT inhibited ferroptosis-dependent intestinal I/R injury. Compared to the I/R + CAT group, total GSH and the total GSH/GSSG ratio decreased (Figure 7(c-d)), while the MDA and Fe^2+^ levels increased in the CAT + JNJ-treated group (Figure 7(e-f)). In addition, the mRNA and protein levels of Gpx4 and Fth1 were downregulated, while the mRNA and protein levels of Cox-2 were upregulated by JNJ during intestinal I/R (Figure 7(g-j), Fig. S4D-F). Compare with I/R + CAT group, CAT + JNJ treatment significantly increased the intestinal tissue HE pathological damage scores and reduced mouse survival (Figure 7(k-l)). Furthermore, CAT treatment increased the relative mRNA and protein levels of tight junction Occludin and ZO-1 following I/R, which were abolished by JNJ (Figure 7(m-o), Fig. S4G-H).

### CAT enhances Gpx4 expression and inhibits ferroptosis by activating TRPV1 in an intestinal organoid H/R injury model

**Figure 8.**
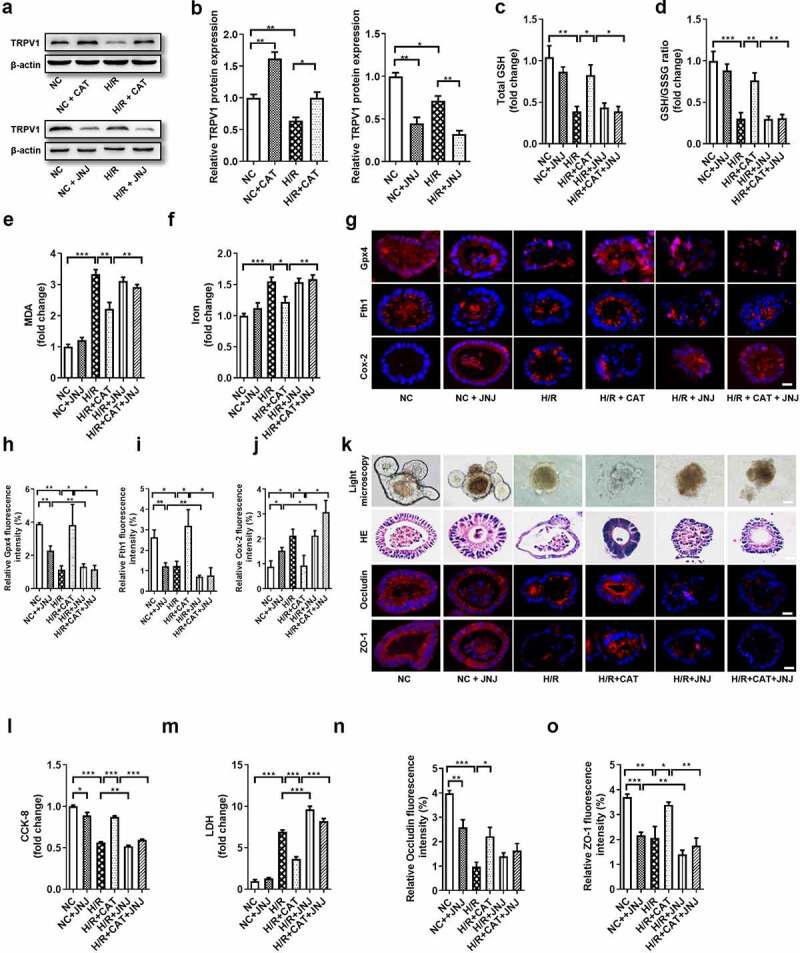
**CAT enhances Gpx4 expression and inhibits ferroptosis by activating *TRPV1* in an intestinal organoid H/R injury model**. (a-b) TRPV1 protein level in the organoids (c-d) Total GSH and the ratio of total GSH/GSSG in the organoids. (e-f) MDA and Fe^2+^ levels in the organoids. (g) The protein levels of Gpx4, Fth1 and Cox-2, scale bar is 20 μm. (h-j) Relative quantitative statistics of Gpx4, Fth1 and Cox-2 protein expression (k) Ileum organoid morphology observed by light microscopy and HE staining and the expression of intestinal barrier tight junction proteins Occludin and ZO-1 in the organoids observed by immunofluorescence, scale bar is 20 μm. (l) Organoid viability measured by CCK-8. (m) Levels of LDH released into the medium. (n-o) Relative fluorescence quantitative statistics of Occludin and ZO-1 protein expression. The results are expressed as the mean ± SEM. n = 6. * *p* < .05, ** *p* < .01, *** *p* < .001 by one-way ANOVA (Tukey’s test)

JNJ was likewise used to explore the role of *TRPV1* in the protective ability of CAT against organoid H/R-induced ferroptosis and injury. The mRNA and protein levels of *TRPV1* were upregulated by CAT, but were downregulated by JNJ (Figure 8(a-b), Fig. S4I). Consistent with the previous results, CAT inhibited ferroptosis-dependent organoid H/R injury. Total GSH and total GSH/GSSG ratio were higher (Figure 8(c-d)), while the MDA and Fe^2+^ levels were lower (Figure 8(e-f)) in the H/R + CAT group than those in the H/R + CAT + JNJ group. Meanwhile, CAT treatment increased the mRNA and protein levels of Gpx4 and Fth1 and decreased the mRNA and protein levels Cox-2, which were abolished by JNJ (Figure 8(g-j), Fig. S4J-L). Small intestinal organoid H/R-induced injury were milder in the H/R + CAT group than in the H/R + CAT + JNJ group, as indicated by light microscopy and HE staining (Figure 8k). The organoids vitality increased, while the LDH levels in the medium were reduced in the H/R + CAT group compared with the H/R + CAT + JNJ group (Figure 8(l-m)). Furthermore, CAT treatment increased the relative mRNA and protein levels of tight junction Occludin and ZO-1, which were abolished by JNJ (Figure 8(k, n-o); Fig. S4M-N).

### CAT is negatively correlated with patient intestinal I/R damage

**Figure 9.**
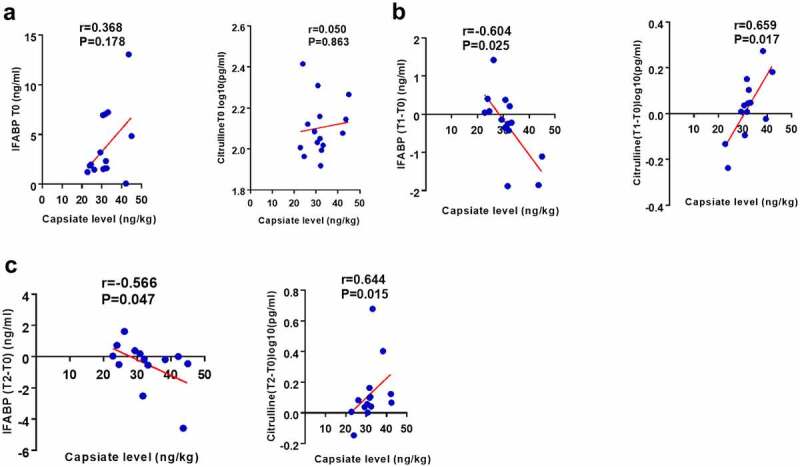
**CAT is negatively correlated with postoperative intestinal injury in patients undergoing cardiopulmonary bypass**. (a-c) The correlation analysis between the levels of CAT in the patient’s pre-operative stool and the levels of intestinal fatty-acid binding protein (IFABP), and citrulline, in plasma preoperative (T0), 6 hours (T1) or 12 hours (T2) after surgery. The results are expressed as the mean ± SEM.* *p* < .05, ** *p* < .01, *** *p* < .001 by spearman analysis

We collected stool and blood samples pre-operation (T0), and at 6 h (T1) and 12 h (T2) after operation. Results of the correlation analysis showed that there is no significant correlation between the CAT content in patient’s pre-operative stool and preoperative plasma levels of citrulline, a negative biomarker of intestinal I/R injury, and intestinal fatty-acid binding protein (IFABP), a positive biomarker of intestinal I/R injury (Figure 9a). While the CAT content in the patient’s preoperative stool positively correlated with the plasma citrulline content at T1 and T2, and negatively correlated with the plasma IFABP content at T1 and T2 (Figure 9 (b-c)).

## Discussion

In this study, we firstly found that CAT is present in the contents of the cecum and is a metabolite of the gut microbiota. Furthermore, we demonstrated that the CAT level in the preoperative stools of patients undergoing CPB is negatively correlated with IFABP, a positive marker of intestinal I/R injury, in patient sera at 6 and 12 hours after surgery. Preoperative CAT levels in the stool were also positively correlated with citrulline, a negative marker of intestinal I/R injury, at 6 and 12 hours after surgery. These indicate that the gut microbiota metabolite CAT is a potentially reliable drug for treating intestinal I/R injury. In addition, we uncovered that ferroptosis is present in intestinal I/R injury in vivo and in ileum organoid H/R injury. We found that promoting ferroptosis intensifies, while inhibiting ferroptosis decreases intestinal I/R injury in vivo and in vitro. Meanwhile, we revealed that CAT promotes Gpx4 expression and reduces ferroptosis-dependent intestinal I/R damage or organoid H/R injury by activating *TRPV1*. Taken together, these results suggest CAT, a metabolite of the gut microbiota, is a potential drug for the treatment of ferroptosis-dependent intestinal I/R injury.

Compared with simple intestinal epithelial cell lines, organoids have the physiology of natural intestinal epithelium (including not only intestinal epithelial cells, but also endocrine cells, Paneth cells, goblet cells, etc.), 3D structure and functional diversity.^24^ The mechanisms and treatments of diseases and injuries provide powerful new models. Compared with experiments in *vivo*, organoids experiment in *vitro* avoid the interference of multiple complex factors in vivo and are more convenient and accurate. Organoids have been chosen as Method of the Year 2017, for their immense potential as tools to study human biology in health and disease. In this study, we have established an intestinal organoid H/R model to simulate intestinal I/R injury and have confirmed that the intestinal organoid is a reliable in vitro model for studying the mechanism and treatment of intestinal I/R injury.

A group has uncovered that CAT affects intestinal fatty acid intake.^25^ In addition CAT administration was suggested to improve glucose tolerance, prevent obesity and hepatic steatosis, and improve bowel changes caused by a high-fat diet.^26^ Zhang et al. found that CAT can reduce the blood sugar level of rats with streptozotocin-induced diabetes by increasing rat insulin level and glycogen content, but the effect was weaker than that of capsaicin.^18^ These studies showed that CAT can promote energy consumption and metabolism, inhibit body fat accumulation, and have antioxidant, anti-inflammatory and anti-tumor activities. However, the role of CAT in intestinal I/R and the relationship between CAT and ferroptosis have not yet been elucidated. In this study, we not only firstly confirmed that CAT is a metabolite of the gut microbiota, but also confirmed for the first time that the CAT content in the preoperative feces is negatively correlated with the postoperative intestinal I/R injury level and gastrointestinal complication score of CPB patients. In addition, we also confirmed for the first time that CAT can reduce intestinal I/R injury and organoid H/R injury and have revealed its novel mechanism of reducing intestinal I/R injury in the context of ferroptosis. Furthermore, we are the first to report that CAT inhibited ferroptosis-related intestinal I/R injury and organoid H/R injury by promoting Gpx4 expression. We are also the first to suggest that CAT enhances Gpx4 expression and inhibits ferroptosis by activating TRPV1 in intestinal I/R injury and organoid H/R injury. Therefore, our research has confirmed the source of CAT in vivo, its potential clinical applications, and the mechanisms underlying its role in intestinal I/R injury. CAT and capsaicin have similar structures and are both TRPV1 agonists, some group found that capsaicin leads to downregulation of TRPV1 at the protein level by inducing internalization and degradation.^27^ However, Zhang et al. revealed that CAT and capsaicin both increased TRPV1 protein expression;^18^ Liang et al. also showed that capsaicin increased the TRPV1 protein expression in jejunum, ileum and colon, while novobiocin, a Newly Found TRPV1 Inhibitor, decreased the TRPV1 protein expression.^28^ In this study, we found that CAT promoted, while JNJ inhibited the TRPV1 expression during intestinal I/R.

Ferroptosis is a newly discovered type of programmed cell death and is distinct from apoptosis, necroptosis, pyroptosis, and autophagic cell death. Through the iron-dependent phospholipid peroxidation process, the cell membrane is damaged, thereby inducing cell death. Dong et al. have uncovered that inhibiting ferroptosis by regulating SLC7A11 helps improve intestinal I/R-induced lung injury.^29^ Consistent with this, Li et al. found that inhibiting ferroptosis can reduce acute lung injury caused by intestinal I/R.^3^ In this study, the connection between CAT and ferroptosis was established for the first time, providing a novel potential mechanism for CAT protectivity against intestinal I/R injury. Furthermore, our study has revealed, for the first time, that the gut microbiota and its metabolites regulate intestinal I/R-induced ferroptosis.

However, this study still has some limitations. CAT is a metabolite of gut microbiota, but it is not completely clear which specific strains can produce CAT; and how these CAT-producing flora changes in intestinal I/R and whether it can reduce intestinal I/R damage has not yet been elucidated. Although we confirmed that there is no obvious correlation between the CAT level in the cecum and the levels of Verrucomicrobia, the mice in this study have high levels of Verrucomicrobia, which may affect the metabolites detected in the untargeted metabolomics analysis. In this study, we found that CAT promotes GPX4 expression and inhibits iron death by activating *TRPV1*, but the specific mechanism of how activated TRPV1 affects GPX4 expression during intestinal I/R injury has not yet been elucidated. Considering that this is not the focus of our research, and it does not affect the reliability of the conclusions, we will further explore this issue in future research. In this study, we detected the overall oxidative stress level and lipid peroxidation level (GSH and MDA) of intestinal tissues and organoids, but the fatty acid content of lipid ROS, which can be altered by CAT, is an important component of oxidative stress level and lipid peroxidation level were still unkown. In addition, RSL3 has poor in vivo bioavailability, and RSL3 can only partially reduce, but cannot completely inhibit, the expression and activity of Gpx4 in this study.

Taken together, the findings of this study indicate that CAT is a metabolite of the gut microbiota and displays an ability to protect the intestine from I/R injury. To our knowledge, we are the first to report that this protective capacity involves the enhancement of Gpx4 through the activation of *TRVP1*, thereby inhibiting ferroptosis. The findings of this study provide potential avenues for treating intestinal I/R injury.

## Materials and methods

### Animals

Six- to eight-week-old specific pathogen-free male C57BL/6 mice were purchased from the animal center of Nanfang Hospital of Southern Medical University (Guangzhou, China). All mice were housed under controlled temperature and humidity conditions, with a 12-hour light-dark cycle, and had free access to food and water. The mice were fasted overnight before the experiment. All experimental procedures were carried out in accordance with the National Institutes of Health guidelines and were approved by the local Animal Care and Use Committee of the Nanfang Hospital of Southern Medical University.

## Intestinal I/R mouse model

The mouse model for intestinal I/R injury was established as in our previous study.^30^ Briefly, the mice were anesthetized with isoflurane. A noninvasive microvascular artery clip was placed on the superior mesenteric artery (SMA) for 60 min, and the clip was removed for reperfusion for 2 hours. During the study period, body temperature was maintained at 37°C with a heating pad, and liquid resuscitation was performed by subcutaneous injection with 0.5 ml of physiological saline immediately after reperfusion.

## Extraction and culture of organoids and the establishment of hypoxia-reoxygenation (H/R) models *in vitro*

The extraction and culture of small intestinal organoids was performed as previously described.^31^ The separated intestinal crypts are fixed onto the bottom of the dish with Matrigel (STEMCELL Technologies Inc., Shanghai, China) drops and covered with IntestiCult medium (STEMCELL Technologies Inc.).

For the establishment of the organoid H/R model, the organoids were placed in a humid, anaerobic environment at 37°C for 12 hours and then placed in an aerobic environment containing 5% CO_2_ in a 37°C incubator for 4 hours.

### Experimental design

The mice and organoids were divided into the following groups. Except that the number of experimental mice with survival rate is n = 20, the other mice experiment n = 8; the number of all organoids experiments is n = 6. The investigator who established the intestinal I/R or H/R model is not clear about the pretreatment and group allocation.

To observe the role of ferroptosis played in the intestinal I/R injury *in vivo*, the mice were randomly divided into 4 groups (Fig. S1A). (1) sham group: except for not performing I/R operation, all other operation steps are the same as intestinal I/R group; (2) Sham + ferrostatin-1 (Fer): the mice were intraperitoneally injected with 10 mg/kg ferroptosis inhibitor ferrostatin-1 (Fer) (MedChemExpress) for 4 hours; (3) I/R group; (4) I/R + Fer: the mice were intraperitoneally injected with 10 mg/kg ferroptosis inhibitor Fer 1 hour before intestinal I/R in mice. To observe the role of ferroptosis played in the intestinal I/R injury *in vitro*, the organoids were randomly divided into 4 groups (Fig. S1B). (1) normal control (NC) group: except for not performing H/R operation, all other operation steps are the same as H/R group; (2) H/R + Fer: 500 nM Fer was added to the organoids for 17 hours; (3) H/R group; (4) H/R + Fer: 500 nM Fer was added to the organoids 1 hour before H/R.

To explore the protective effect of CAT on intestinal I/R *in vivo*, the mice were randomly divided into 4 groups (Fig. S2E). (1) Sham group; (2) Sham + CAT group: the mice were intraperitoneally injected with 2 mg/kg CAT (Alomone Labs, Shanghai, China) for 4 hours; (3) I/R group; (4) I/R + CAT group: the mice were intraperitoneally injected with 2 mg/kg CAT 1 hour before intestinal I/R in mice. To explore the protective effect of CAT on intestinal I/R *in vitro*, the organoids were randomly divided into 4 groups (Fig. S2F). (1) NC group; (2) NC + CAT group: 100 μM CAT was added to the organoids for 17 hours; (3) H/R group; (4) H/R + CAT group: 100 μM CAT was added to the organoids 1 hour before H/R.

To explore the role of Gpx4 played on the protective effect of CAT in intestinal I/R *in vivo*, the mice were randomly divided into 6 groups (Fig. S3A). (1) Sham group; (2) Sham + RSL3 group: the mice were intraperitoneally injected with 30 mg/kg Gpx4 inhibitor RSL3 (MedChemExpress) for 4 hours; (3) I/R group; (4) I/R + CAT group; (5) I/R + RSL3: the mice were intraperitoneally injected with 30 mg/kg Gpx4 inhibitor RSL3 1 hour before intestinal I/R in mice; (6) I/R + CAT + RSL3 group: the mice were intraperitoneally injected with 30 mg/kg RSL3 and 2 mg/kg CAT 1 hour before intestinal I/R in mice. To explore the role of Gpx4 played on the protective effect of CAT in intestinal I/R *in vitro*, the organoids were randomly divided into 6 groups (Fig. S3B). (1) NC group; (2) NC + RSL3 group: 5 μM RSL3 was added to the organoids for 17 hours; (3) H/R group; (4) H/R + CAT group; (5) H/R + RSL3: 5 μM RSL3 was added to the organoids 1 hour before H/R; (6) H/R + CAT + RSL3 group: 5 μM RSL3 and 100 μM CAT was added to the organoids 1 hour before H/R.

To explore the role of *TRPV1* played on the protective effect of CAT in intestinal I/R *in vivo*, the mice were randomly divided into 6 groups (Fig. S4A). (1) Sham group; (2) Sham + JNJ-17203212 (JNJ) group: the mice were intraperitoneally injected with 40 mg/kg selective *TRPV1* antagonist JNJ (MedChemExpress) for 4 hours; (3) I/R group; (4) I/R + CAT group; (5) I/R + JNJ group: the mice were intraperitoneally injected with 40 mg/kg selective *TRPV1* antagonist JNJ 1 hour before intestinal I/R in mice; (4) I/R + CAT + JNJ group: the mice were intraperitoneally injected with 40 mg/kg JNJ and 2 mg/kg CAT 1 hour before intestinal I/R in mice. To explore the role of *TRPV1* played on the protective effect of CAT in intestinal I/R *in vitro*, the organoids were randomly divided into 6 groups (Fig. S4B). (1) NC group; (2) NC + JNJ group: 0.5 μM JNJ was added to the organoids for 17 hours; (3) H/R group; (4) H/R + CAT group; (5) H/R + JNJ: 0.5 μM JNJ was added to the organoids 1 hour before H/R; (6) H/R + CAT + JNJ group: 0.5 μM JNJ and 100 μM CAT was added to the organoids 1 hour before H/R.

## Patient samples

Due to the hypoperfusion of intestinal blood flow and hypoxia caused by cardiopulmonary bypass, patients who need cardiopulmonary bypass (CPB) surgery were used as cases for collecting intestinal I/R samples as previously described.^32,33^ From 2019 July to 2020 January, we recruited consecutive patients who underwent elective cardiac valve replacement or coronary artery bypass graft under CPB at the Department of Cardiac Surgery, in Southern Medical University Nanfang Hospital, Guangzhou, China. Participants were not included if they (1) were <18 or >75 years old, (2) had chronic kidney disease, (3) had chronic digestive system diseases, previous gastrointestinal surgery, or confirmed or suspected intestinal ischemia/necrosis, and (4) used antidiarrheals, laxatives or prebiotics within one week, or used antibiotics within 3 months.

Finally, a total of 20 patients were enrolled. Blood and fecal samples were collected from the 20 patients before surgery. The study protocol was approved by the Ethical Committee of Nanfang hospital, Southern Medical University (approval number NFEC-202009-k2-01). All individuals gave informed consent to participate.

Blood samples were collected preoperatively (T0) and at 6 h (T1) and 12 h (T2) after surgery in EDTA plasma tubes as well as in serum separator tubes for analyses of intestinal fatty-acid binding protein (IFABP) and citrulline, respectively. The level of IFABP in plasma is a confirmed positive marker,^34^ while citrulline is a reliable negative biomarker for predicting and diagnosing intestinal I/R injury.^35^ Fecal samples were collected preoperatively, and the levels of CAT were quantified by liquid chromatograph-tandem mass spectrometry (LC-MS/MS). IFABP and citrulline in the plasma samples were measured using a human IFABP ELISA Kit (Bio-Swamp, Wuhan, China) and citrulline ELISA Kit (USCN, Wuhan, China), respectively, at multiple time points (T2-T0) to determine concentration differences. The detection of CAT, IFABP, and citrulline were performed by researchers blinded to the group allocation.

## Detection of organoid injury by CCK-8 and lactate dehydrogenase (LDH) assays

The CCK-8 kit (Dojindo, Shanghai, China) was used to detect cell viability and the LDH kit (Nanjing Jiancheng Bioengineering Institute, Nanjing, China) was used to detect the level of LDH in the culture medium to assess organoid damage. The detection of CCK-8 and LDH was carried out based on the manufacturers’ protocols.

## DNA extraction and qPCR analysis

Cecum contents were collected after intestinal I/R and stored at −80°C until DNA extraction. They were resuspended in 0.5 ml phosphate-buffered saline (PBS) containing 0.5% Tween 20, vortexed gently, and then freeze-thawed at −80°C and 60°C three times to disrupt cellular membranes. Microbial DNA from cecum samples were then extracted using the classical phenol-chloroform extraction method as previously described.^20^ All extracted DNA was stored at −20°C until further use. The extracted fecal DNA was diluted to 10 ng/μl, and quantitative real-time PCR (qPCR) was performed using 16S rRNA primers, and Bacteroidetes primers. The primers are listed in the Supplementary data.

## 16S rRNA gene sequencing

After DNA extraction, the V4 region of 16S rRNA gene was amplified using specific barcode primers (V4F, 5ʹ-GTGTGYCAGCMGCCGCG GTAA-3ʹ and V4R, 5ʹ-CCGGACTACNVGGG TWTCTAAT-3ʹ). The PCR amplification products were mixed in equal amounts measured by QuantiFluor. All samples were subjected to paired-end sequencing on the Illumina Hiseq PE250 (San Diego, CA, USA) platform. High-throughput sequencing analysis of bacterial rRNA genes was processed using the Quantitative Insights into Microbial Ecology (QIIME, version 1.9.1) software suite.^36^ Low-quality reads were filtered after quality control, and the remaining high-quality reads were assigned to operational taxonomic units (OTUs) with ≥97% similarity using the UPARSE pipeline. Representative sequences for each OTU were classified into organismsusing the RDP classifier (version 2.2) based on the SILVA Database (https://www.arb-silva.de/). QIIME was applied to analyze the alpha and beta diversities, based onweighted and unweighted UniFrac distances successively. The Metastats (version 20090414) and LEfSe (version 1.0) softwares were used to explore biomarker features in each group. Kyoto Encyclopedia of Genes and Genomes (KEGG) pathway analysis of the OTUs was performed using Tax4Fun (version 1.0) and was performed using the OmicShare tools, a free online platform for data analysis (www.omicshare.com/tools). The calculated *p*-value was gone through FDR Correction, taking FDR ≤ 0.05 as a threshold.

## Fecal metabolic profiling

The nontargeted metabolomics procedure was performed by ESI-QTOF/MS (Xevo 121 G2-S Q-TOF, Waters) and UPLC-QTOF/MS (ACQUITY UPLC I-Class, Waters) as described previously.^37^ Briefly, cecum samples (100 mg) were dissolved in 500 μL of ice-cold water, mixed using a vortex, and centrifuged for 15 min at 12,000 r/min. Then, the supernatant was obtained, and the remaining precipitate were further extracted with 500 μL of ice-cold methanol. The two fecal extracts were combined and centrifuged at 12,000 rpm for 15 min and the supernatant was stored at 4°C; 10 μL of the supernatant was used for analysis. The MS data of cecum samples were first processed by MarkerLynx (version 4.1, Waters, Milford, MA, USA). The procedure included integration, normalization, and peak intensity alignment. In the positive data set, a list of m/z and retention time with corresponding intensities was provided for all metabolites in every sample. Then, the processed data set was then entered into the SIMCA-P software package (v13.0, Umetric, Umeå, Sweden). The normalized data were then used to perform principal component analysis (PCA) and orthogonal to partial least squares-discriminate analysis (OPLS-DA) with VIP>1 as a threshold.

Targeted metabolomics (capsiate measurement) was performed by LC-MS/MS as described previously.^38^ Briefly, cecum samples (100 mg) were dissolved in 900 μL of ice-cold water and were extracted via sonication in water for 10 min. After ethyl acetate was added, shook for 3 min, the samples were centrifuged at 13000 rpm at 4°C for 10 min. The supernatant was collected and dried with nitrogen, and then reconstituted with methanol/ammonium acetate pH4.5 (60:40 v/v) for further computer analysis. The chromatographic separation was performed on the Thermo Scientific Prelude SPLC system, and detection was performed on the Thermo TSQ Vantage triple quadrupole mass spectrometer. Data collection and processing were performed with TraceFinderTM software version 3.3 sp1 (Thermo Fisher Scientific Corp., USA)

## Hematoxylin-eosin staining

Ileum samples tissue were collected and fixed in 4% paraformaldehyde. Then, the samples were embedded in paraffin; 5-μm-thick sections were created and stained with hematoxylin-eosin (HE) according to the standard protocol. Images were captured at 200× with an Olympus fluorescence microscope (Olympus, Tokyo, Japan). The pathological scores of intestinal mucosal injury were evaluated by blinded technicians, and were grouped according to the Chiu scoring system.^39^

## Immunofluorescence and immunohistochemistry

Immunofluorescence and immunohistochemistry were performed as previously described.^30^ Anti-zona occludens 1 (ZO-1) antibody (ab216880, Abcam, Cambridge, MA, USA), anti-Occludin antibody (ab216327, Abcam), anti-ferritin heavy chain 1 (Fth1) antibody (ab183781, Abcam), anti-Gpx4 antibody (ab125066, Abcam), and anti-Cox-2 antibody (ab179800, Abcam) were used to detect protein expression in the intestinal tissue and organoids. Images were captured at 200× with an Olympus immunofluorescence microscope. Quantification of the relative intensity of protein staining was performed by automated image analysis in five randomly chosen 200× fields for each sample.

## RNA extraction and RT-PCR

RNA was extracted with the TRIzol reagent (Invitrogen, New York, USA). Real-time PCR was performed using the ABI Q5 Real-Time PCR System (Applied Biosystems, Foster City, CA, USA), with the SYBR Green detection protocol (TOYOBO, Tokyo, Japan). The expression of target genes in mice was normalized against that of the housekeeping gene 18S using the 2^−ΔΔ^CT method. The target gene primers are shown in Supplementary Table S2.

## Glutathione (GSH), lipid peroxidation, and ferrous ion (Fe^2+^) assays

The concentrations of GSH, malondialdehyde (MDA), and Fe^2+^ in tissues or tissue lysates were detected using the Iron Assay Kit (Abcam), MDA Assay Kit (Abcam), and GSH Assay Kit (Abcam), respectively. All kits were used according to the manufacturer’s instructions.

## Western blot

The protein expression of TRPV1 were detected by western bolt. The RIPA lysis buffer (Solarbio) was used to extract total protein from cells. The extracted protein was separated by 10% SDS-PAGE gel electrophoresis, then transferred to a PVDF membrane, blocked PVDF with 5% skimmed milk powder for 1 hour, and then incubated with TRPV1 (ab203103, Abcam) antibodies at 4°C overnight. The next day, the PVDF membrane was cleaned three times with TBST (Solarbio) for 5 minutes each time. After incubating the corresponding secondary antibody at room temperature for 2 hours, the PVDF membrane was cleaned three times with TBST for 5 minutes each time again. Observing protein bands using enhanced chemiluminescence (Thermo Fisher Scientific, Inc.).

## Statistical analysis

Data were analyzed and performed using GraphPad Prism software (version 7.0) by investigators blinded to the group allocation. The results are expressed as the mean ± SEM. Means of 2 continuous normally distributed variables were compared by independent samples Student’s t-test. The Mann-Whitney U test and the Kruskal-Wallis test were used, respectively, to compare the means of 2 and ≤ 3 groups of variables that were not normally distributed. In addition, the Spearman method was used for correlation statistical analysis. A value of *p* < .05 was considered significant.

## Supplementary Material

Supplemental Material

## Data Availability

The raw sequencing data generated from this study have been deposited in NCBI (Sequence Read Archive) SRA (http://www.ncbi.nim.nih.gov/sra) under the accession number SRP287561.
